# Materia Medica and the Medical Curriculum

**Published:** 1903-10-31

**Authors:** 


### Materia Medica and the Medical Curriculum.

It is the natural habit of a professor to exalt to a high level in the scheme of education the particular subject which it is his duty to teach. Whatever else goes by the board, room must at all costs be found for his special contribution and influence. Such a disposition, not by any means an entirely blameworthy one, is probably responsible for a recent pronouncement that materia medica and pharmacy ought to be excluded from the medical curriculum and left to those who desire a pharmaceutical diploma. It may at once be allowed that a commercial knowledge of drugs, an exact acquaintance with their histology, and a capacity to recite their several botanical and geographical sources, are of little or no value to the student of medicine. These are departments of knowledge which lie within the province of the pharmacist. Again, there is no difficulty in admitting that the recent developments of pharmacology are matters of great importance, and that no scheme of medical training which excludes or minimises these can be regarded as satisfactory. These statements, however, do not involve assent to the suggestion that the raw materials of pharmacy are of no value to the medical student. They acknowledge that there are departments of materia medica which may be wisely left to the commercial and manufacturing pharmacist. But they also imply that there are other aspects of the subject which come within the domain of medical study. The line of distinction can be drawn with considerable confidence if it is recognised that the physician is concerned with the use of medicines as remedies whilst the work of the pharmacist is merely the purchase and sale of drugs and the manufacture of their preparations. There are several reasons why materia medica and pharmacy should claim a due measure of attention from the student of medicine. In the first place he will as a practitioner have to prescribe drugs, and surely in doing so he ought to know these as facts and not merely as names. If all knowledge of crude drugs is to be put outside the medical curriculum the student may escape the modest claims of the materia medica museum, but his position in relation to the terms of his prescriptions will be a singularly unsatisfactory one. Again, for purposes of treatment, drugs must be presented as preparations, and must be suitably combined and dispensed. It is notorious that even under existing conditions the student often leaves his school very inadequately instructed in these respects. A proposal to diminish further his already scanty opportunities is not in the interests of professional efficiency. One other consideration may also be mentioned. The medical practitioner inevitably comes into more or less close relationship with the trained and educated pharmacist. It does not conduce to the dignity of the profession that the physician should show himself to be practically ignorant of the properties of the medicines he employs and of the methods by which they are prepared. It is unfortunate when a demonstration of such ignorance justifies what an old writer terms "the sneering of the apothecary's boy." These are some of the reasons which demand the inclusion of materia medica and pharmacy within the borders of the medical curriculum. Unfortunately there are some medical schools which proceed on the assumption that these subjects can be taught by anyone, and thus the most inexperienced member of the staff may succeed to a lectureship for which he has never cultivated either ambitions or qualifications. The results are well known. What is to be desired for the medical student is a practical acquaintance with the physical and chemical properties of medicines, and a general knowledge of the principles of pharmaceutical processes. To this, by the discipline of pharmacology and therapeutics, he must add the ability to apply medicines as practical remedies. But to such discipline he ought to come possessing actual and not merely nominal knowledge of the materials to be employed. This knowledge he can only gain by a practical training in materia medica and pharmacy. A well-organised medical school ought to provide this training in the shape of a museum, a pharmaceutical workshop, and an efficient teacher. If it cannot do this it should refer the student either to a school of pharmacy or to the shop of a pharmacist. In many of the latter three months' diligent work will secure a knowledge of drugs the value of which can hardly be overstated.

